# Activation of Store-Operated Calcium Entry in Airway Smooth Muscle Cells: Insight from a Mathematical Model

**DOI:** 10.1371/journal.pone.0069598

**Published:** 2013-07-25

**Authors:** Huguette Croisier, Xiahui Tan, Jose F. Perez-Zoghbi, Michael J. Sanderson, James Sneyd, Bindi S. Brook

**Affiliations:** 1 School of Mathematical Sciences, University of Nottingham, Nottingham, United Kingdom; 2 Department of Microbiology and Physiological Systems, University of Massachusetts Medical School, Worcester, Massachussetts, United States of America; 3 Department of Cell Physiology and Molecular Biophysics, Texas Tech University Health Sciences Center, Lubbock, Texas, United States of America; 4 Department of Mathematics, University of Auckland, Auckland, New Zealand; University of Debrecen, Hungary

## Abstract

Intracellular 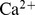 dynamics of airway smooth muscle cells (ASMC) mediate ASMC contraction and proliferation, and thus play a key role in airway hyper-responsiveness (AHR) and remodelling in asthma. We evaluate the importance of store-operated 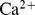 entry (SOCE) in these 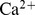 dynamics by constructing a mathematical model of ASMC 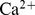 signaling based on experimental data from lung slices. The model confirms that SOCE is elicited upon sufficient 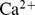 depletion of the sarcoplasmic reticulum (SR), while receptor-operated 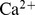 entry (ROCE) is inhibited in such conditions. It also shows that SOCE can sustain agonist-induced 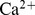 oscillations in the absence of other 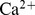 influx. SOCE up-regulation may thus contribute to AHR by increasing the 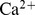 oscillation frequency that in turn regulates ASMC contraction. The model also provides an explanation for the failure of the SERCA pump blocker CPA to clamp the cytosolic 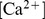 of ASMC in lung slices, by showing that CPA is unable to maintain the SR empty of 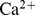. This prediction is confirmed by experimental data from mouse lung slices, and strongly suggests that CPA only partially inhibits SERCA in ASMC.

## Introduction


**Figure 1:**
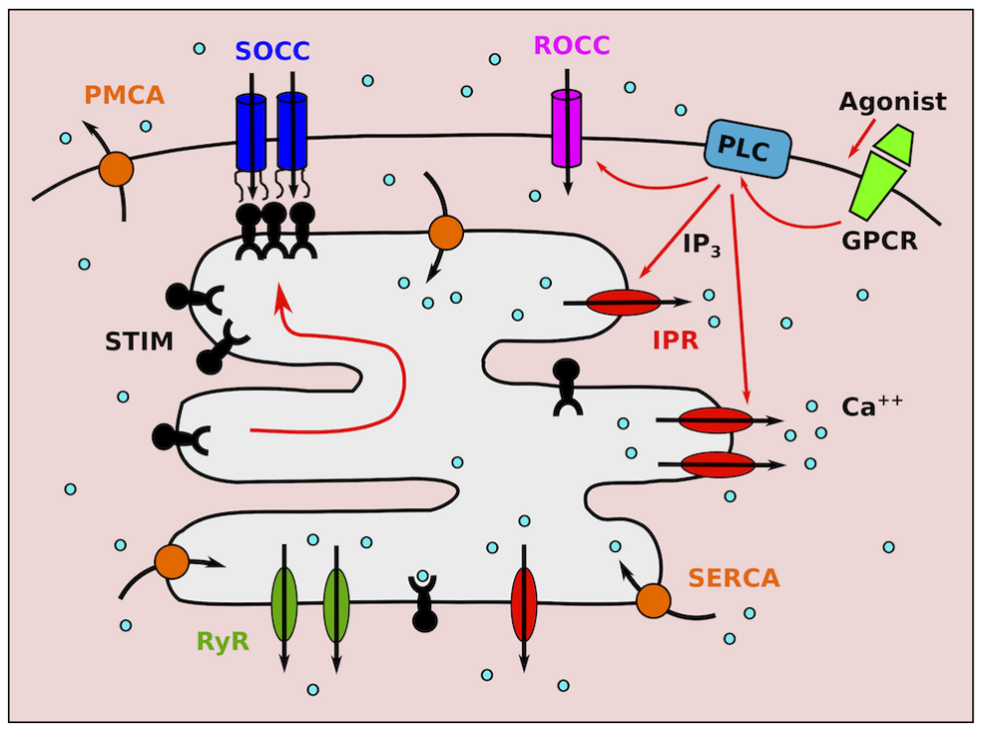
Schematic of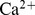 signalling in ASMC. Agonist stimulation of G-protein coupled receptors (GPCR) induces PLC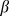 activation, giving rise to 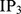 production and 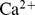 entry through receptor-operated 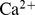 channels (ROCC). 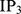 triggers 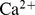 release through IPR. Depletion of the SR from 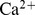 causes STIM protein oligomerisation and migration toward the cell membrane, where they bind and activate store-operated 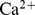 channels (SOCC). 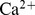 ATP-ases pump 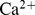 back into the SR (SERCA) and out of the cell (PMCA).

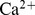
 is a ubiquitous cellular messenger, controlling a wide range of biological functions. These include ASMC contraction and proliferation, which are associated with airway hyper-responsiveness (enhanced contractility) and airway remodelling (structural changes) in asthma. The main trigger for cytoplasmic 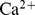 (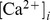) increase in ASMC is agonist stimulation at the cell membrane (e.g., by histamine released from mast cells or acethylcholine released from nerves). Binding of agonist to G-protein coupled receptors induces the production of 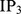, a second messenger which diffuses into the cytosol and binds to 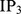 receptor 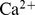 channels (IPR) on the sarcoplasmic reticulum (SR) membrane (Fig. 1). This causes the IPR to open and release 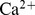 from the SR into the cytosol (the SR being the main 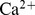 store in ASMC). As 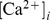 exerts a positive feedback on IPR, this results in so-called 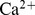 -induced 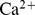 release (CICR). The release is terminated by the inhibition of the IPR at large 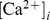, and 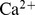 is pumped back into the SR by 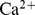 ATP-ases (SERCA). Hence, for sufficient 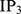 concentration, cycling of 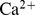 through IPR can occur, and give rise to the repetitive propagation of 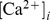 waves through the cytosol. These appear as 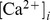 oscillations at the whole-cell level. Importantly, airway contraction increases with the frequency of these 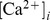 oscillations [1], [2]. 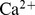 dynamics are also involved in ASMC proliferation [3]–[5], and in the assembly of myosin thick filament and actin thin filament [6]–[8], which form the contractile machinery of ASMC. In addition, several 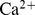 channels and pumps in ASMC are regulated by inflammatory mediators present in asthma (e.g., [4], [9]–[12]). 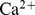 dynamics therefore appear to be involved in multiple interrelated aspects of asthma at the cellular level. In the present work, we use mathematical modelling to investigate the important 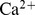 pathways at play in 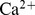 dynamics of ASMC and thus improve our understanding of airway hyper-responsiveness and remodelling in asthma.

Store-operated 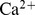 entry (SOCE) is one important 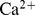 entry mechanism, in which plasma membrane (PM) 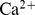 channels open in response to 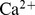 store depletion. These are called store-operated 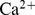 channels (SOCC). Although the concept of SOCE was proposed 25 years ago [13], the mechanism of its activation has been identified only recently [14]. The process is mediated by stromal interaction molecules (STIM), proteins embedded in the SR membrane which are sensitive to SR 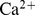. Upon dissociation of 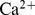 from their SR binding site, they oligomerise and translocate within the SR membrane to the plasma membrane. Here, STIM proteins bind to Orai and/or TRP, the proteins forming the pore of SOCC, and trigger their opening (Fig. 1). Although SOCE has been identified in many cells, it is generally stimulated by artificial emptying of the 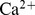 store, as there is unfortunately no specific pharmacological SOCC blocker. Hence, the importance of store depletion, and therefore of SOCE, during physiological conditions such as 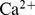 oscillations, remains largely unknown. This may explain why SOCE has been included only in a few mathematical models of 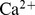 dynamics [15]–[18]. In particular, no prior modelling work on 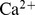 dynamics in ASMC [19]–[23] has taken SOCE into account, even though there is evidence that SOCE is up-regulated by inflammatory mediators found in asthma (TNF-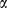 and IL-13) [9], [11], [24], and is associated with ASMC proliferation [3], [5].

In this paper, we develop a mathematical model to evaluate the importance of SOCE in 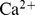 dynamics of ASMC. While there is much evidence that SOCE occurs upon SR depletion in cultured ASMC *in vitro* (e.g., [3], [25]–[27]), these cultured cells often lose their contractile phenotype, and rarely display agonist-induced 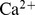 oscillations. Hence, ASMC in lung slices, which retain most of their physiological and morphological characteristics, are a more reliable preparation to study ASMC 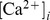 dynamics. Moreover, the available data from lung slices reflect 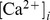 dynamics in individual ASMC, while the majority of works with cultured cells provide only global imaging of 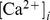 over wells containing thousands of ASMC. Therefore, we base our model on data from lung slices. SOCE has not been studied directly in lung slices, but a treatment with ryanodine-caffeine (Rya-Caf) has previously been used to clamp the cytosolic 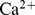 of ASMC [2], [28], [29], which relies on emptying the SR from 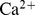. The results of these experiments therefore provide invaluable information about SOCE. Because agonist stimulation was systematically performed before Rya-Caf treatment to ensure that the lung slice is viable, i.e., that ASMC exhibit normal 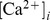 oscillations and contraction, we can construct a mathematical model of 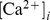 dynamics informed by these data that accounts for both physiological and non-physiological conditions. The model is then used to i) evaluate the effect of SOCE up- and down-regulation on agonist-induced 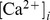 oscillations, and (ii) explain the inability of the SERCA pump blocker CPA to clamp the 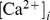, in contrast with Rya-Caf treatment.

## Methods

### Ethics Statement

The experimental study followed the recommendations in the Guide for the Care and Use of Laboratory Animals of the National Institutes of Health. The protocol was approved by the Institutional Animal Care Committee of the University of Massachusetts Medical School (Docket Number: A-836–12). Animals were euthanized with sodium pentobarbital before tissue collection.

### Experimental data

**Figure 2:**
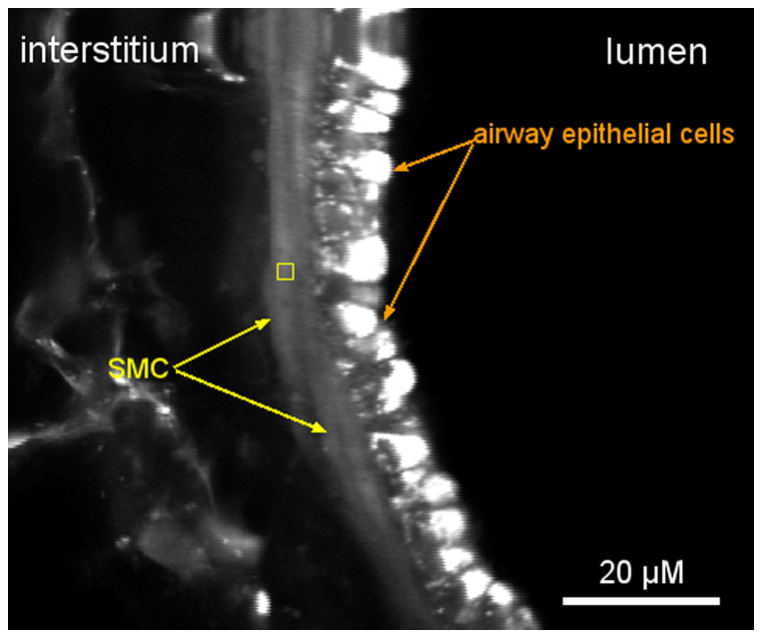
Fluorescence image of part of a mouse airway wall obtained by two-photon laser scanning microscopy. The yellow square shows a typical region, within an ASMC, from which 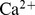 dynamics is imaged.

Data consist of fluorescence recordings of 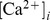 dynamics in ASMC within intact lung slices. All the materials and methods have been previously described (e.g., [2], [28]). Essentially, 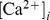 imaging was performed from regions of about 4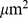 within ASMC (Fig. 2), using two-photon laser scanning microscopy. The fluorescent indicator employed was Oregon Green BAPTA-1-AM, which has a high affinity for 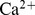 (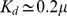
M). We use published data [2] to develop the mathematical model, and new experimental results to test the model predictions (see Results). The latter data can be made freely available upon request for academic, non-commercial use.

### Mathematical model

Intracellular 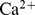 dynamics are modelled at the whole-cell level, via the following system of ordinary differential equations (e.g., [30]):
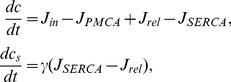
(1)where 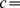 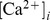 is the free cytosolic 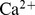 concentration, and 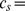 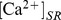 is the free SR 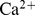 concentration.

The term 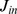 represents the total influx of 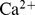 into the cytosol through PM channels; 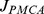, the 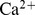 efflux through the PM 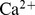 ATP-ase pumps (PMCA); 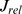, the 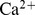 flux of 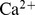 from the SR into the cytosol, and 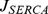, the flux of 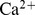 from the cytosol into the SR through the SR/ER 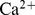 ATP-ases (SERCA). The factor 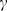 represents the ratio of cytoplasmic volume to SR volume, and implicitly incorporates the relative effect of fast, linear (e.g., low affinity) 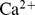 buffers in the SR compared to the effect of similar buffers in the cytosol. Indeed, the effect of fast, linear buffers amounts to a global rescaling of the 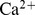 fluxes in the corresponding compartment (e.g., [30]). The other buffers are assumed to have a negligible effect on 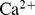 dynamics at the whole-cell level (see also Discussion).

We assume that
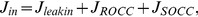
(2)where 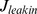 is a constant 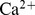 leak through unspecified channels, 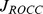 is the 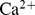 influx through receptor-operated 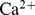 channels (ROCC) and 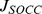 the influx through SOCC. We neglect the 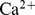 influx through voltage-operated 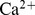 channels (VOCC) because membrane depolarisation plays little role during agonist-induced 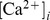 signalling and contraction in ASMC (in contrast to other types of muscle cells, including vascular smooth muscle cells, where action potentials are crucial to contraction) [1], [31]. The 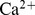 influxes are modelled by:



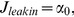
(3a)

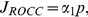
(3b)

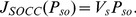
(3c)where 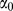 and 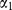 are constants, 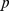 is the agonist concentration, 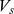 is the maximum SOCC flux, and 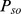 represents the fraction of STIM proteins bound to Orai/TRP proteins, i.e. the fraction of activated SOCC. This fraction adapts slowly to changes in 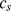, because the diffusion of STIM within the SR membrane is a slow process [32]. We model this phenomenologically by



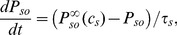
(4a)

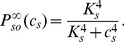
(4b)


The steady-state function 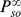 can be interpreted as the fraction of STIM proteins dissociated from SR 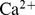 (as a consequence of store depletion), and thus able to oligomerise and move toward the PM to bind with Orai and/or TRP (see also Discussion). 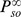 is therefore a decreasing function of 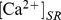, which we model by the reverse Hill function Eq. (0b), assuming affinity 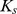 for 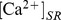 and Hill coefficient 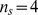 [33].

The total 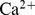 flux from the SR into the cytosol is given by
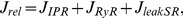
(8)where 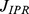 is the 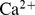 flux through 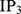 receptors (IPR), 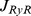 the 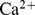 flux through ryanodine receptors (RyR), and 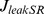 an unspecified 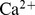 leak out of the SR. We use the formulation (e.g., [30]):
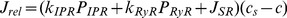
(9)where 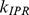 (resp. 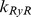) is the maximum rate of 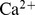 flow through IPR (resp. RyR). Following [23], the IPR opening probability 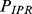 is modelled using the Li-Rinzel/Tang et al. reduction of the De Young-Keizer (DYK) model [34]–[36]:
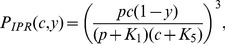
(10)where 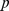 is the 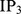 concentration, and 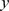 is the fraction of inhibited IPR. The latter obeys



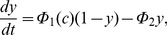
(11)with
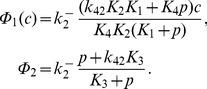
(12)


The parameters 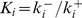 (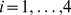) are equilibrium constants for 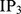 and 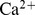 binding/unbinding to the IPR; we use the original values from the DYK model [34]. The value of 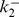 is scaled so that the range of 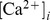 oscillation frequencies matches the experimental range, with the ratio 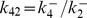 kept constant to the value in ref. [30] (see also Table 1). In the experiments modelled in this work, either RyR play a negligible role, or they are locked open by Rya-Caf treatment (see Results). Hence, we neglect their dynamics and set the fraction of open RyR, 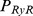, either to 0 or 1 depending on the experiment considered.

**Table 1:** Parameter values used in the model.

Parameter	symbol	value	units	reference
PMCA maximum flux	*V_p_*	7.5	*μ*M/s	this work
PMCA affinity	*K_p_*	1.5	*μ*M	0.1–1 [59]
SOCE maximum flux	*V_s_*	1.57	*μ*M/s	this work
STIM SR Ca^2+^ affinity	*K_s_*	50	*μ*M	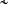 c^*^_s_/2
SOCE Hill exponent	*n_s_*	4		[33]
SOCE timescale	*τ_s_*	30	*s*	[32]
Constant leak influx	*α_0_*	0	*μ*M/s	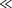 *V_s_*
Cyt/SR vol. × buffer effects	*γ*	5.405		[34], [60]
ROCE rate	*α_1_*	0.00105	*s^−1^*	this work
SERCA maximum flux	*V_e_*	5	*μ*M/s	this work
SERCA affinity	*K_e_*	0.1	*μ*M	0.1–1 [61]
CPA effect timescale	*τ_e_*	30	s	∼ min
IPR rate	*k_IPR_*	0.667	*s^−1^*	this work
Agonist concentration	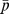	1	*μ*M	this work
Agonist effect timescale	*τ_p_*	30	*s*	∼ min
SR leak rate	*J_SR_*	0.01	*s^−1^*	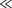 *k_IPR_*
RyR leak rate (Rya-Caf effect)	*K_RYR_*	0.19	*s^−2^*	this work
Rya-Caf effect timescale	*τ_SR_*	10	*s*	∼ min
IPR affinity for 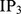	*K_1_*	0.138	*μ*M	[34]
IPR affinity for Ca^2+^ (inhib. site)	*K_2_*	1.05	*μ*M	[34]
IPR affinity for IP_3_	*K_3_*	0.943	*μ*M	[34]
IPR affinity for Ca^2+^ (inhib. site)	*K_4_*	0.144	*μ*M	[34]
IPR affinity for Ca^2+^ (activ. site)	*K_5_*	0.082	*μ*M	[34]
IPR Ca^2+^ dissoc. rate (inhib. site)	*k^−^_2_*	0.167	*s^−1^*	this work
IPR Ca^2+^ dissoc. rate (inhib. site)	*k^−^_4_*	0.138 *k^−^_2_*	*s^−1^*	[34]

For 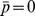 and 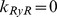, the equilibrium 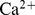 concentrations are 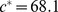
nM and 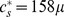
M, which are in the physiological ranges [40], [41].

The 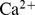 ATP-ases are modelled using the usual expressions (e.g., [30]):
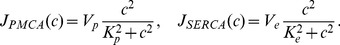
(13)


We do not model 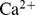 pumping into mitochondria explicitly, but acknowledge that a portion of the extrusion process attributed to PMCA might actually be performed by mitochondria uniporters, as these might be activated at average 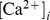 as low as 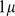
M [20].

Gathering all expressions, the model is described by:
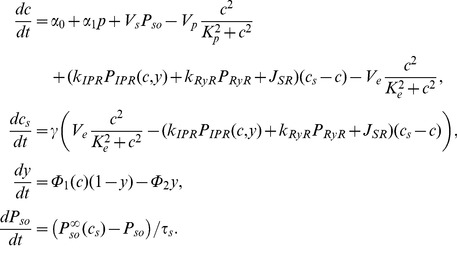
(14)


In addition to Eq. (11), we use the following expressions to account for the time needed by drugs to reach full effect:
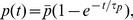
(15)

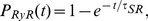
(16)

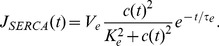
(17)


These equations describe respectively agonist stimulation, Rya-Caf treatment, and SERCA block by CPA (see Results).

Unless otherwise mentioned, parameter values were freely adapted (within physiological ranges when they are known) to account for the experimental results. The values retained are listed in Table 1. The fitting was performed “by hand” (i.e., no algorithmic method was used) within the Mathematica “Manipulate” environment (a useful framework for fitting an ODE model to several experimental results as it enables visualisation of the effect of a parameter change on several ODE integrations almost instantaneously). The code can be made freely available upon request for academic, non-commercial use.

All simulations were run from the same initial condition as in the experiment, which is usually the physiological equilibrium. Bifurcation diagrams were computed using the numerical continuation software AUTO [37], [38].

## Results

### Accounting for 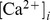 dynamics of AMSC in lung slices

**Figure 3:**
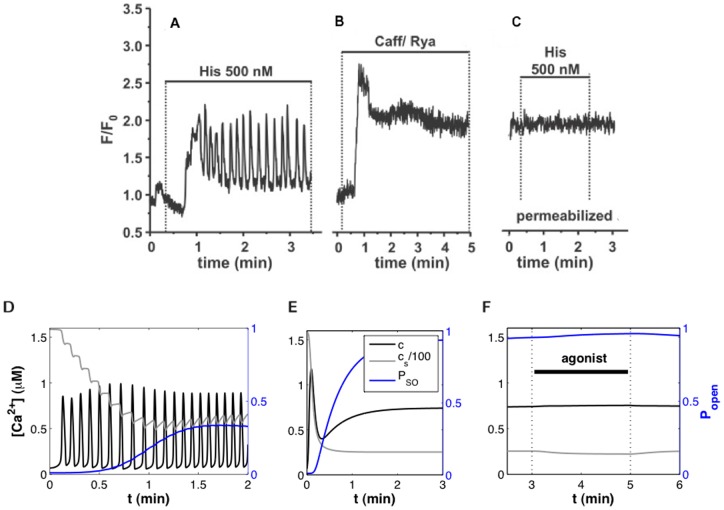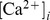 dynamics in ASMC: experiment and model. (A)–(C): Fluorescence imaging of 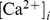 dynamics in an ASMC within a human lung slice, during the following 3-step experiment: (A) Agonist stimulation, (B) Rya-Caf treatment, and (C) second agonist stimulation. Following the irreversible Rya-Caf treatment in (B), agonist stimulation (C) is no longer able to elicit 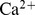 oscillations, nor does it perturb the new elevated 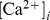 equilibrium. *Reprinted from* [*2*] *under a CC BY license, with permission of the American Thoracic Society, original copyright 2010. Cite: Ressmeyer et al. /2010/Am J Respir Cell Mol Biol/43/179–191. Official journal of the American Thoracic Society. This modified figure is based on the original figure available from* www.atsjournals.org. (D)–(F): Simulations of the experiments in (A)–(C) using Eqs. 114–12 and the parameter values in Table 1. The evolution of 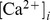, 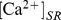, and 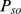 (fraction of open SOCC) are shown (cf. legend in (E)).

Fig. 3A-C shows representative 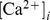 dynamics of an ASMC in a human lung slice in response to a three-step experimental protocol [2]. This protocol was originally designed to clamp the 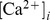 of ASMC, in order to study independently the effects of agonist and 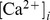 on airway contraction [28]. The slice is first stimulated with agonist (histamine), to verify its viability (Fig. 3A). This induces 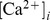 oscillations. Agonist is then washed from the slice, and a Rya-Caf treatment is applied (Fig. 3B). This creates a permanent 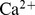 leak through RyR, because caffeine opens RyR and ryanodine locks them open irreversibly. If this 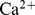 leak is large enough, it keeps the SR empty and prevents any further change in 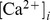, unless extracellular 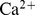 is modified. The effectiveness of the treatment is confirmed by the second application of agonist (Fig. 3C): no further 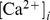 increase is triggered, showing that 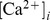 is clamped. It is important to emphasise that these results are not specific to histamine stimulation of human lung slices: similar results have been obtained in mouse and rat lung slices with methylcholine (Fig. 6 in ref. [29], Figs. 5B and 6C-D in ref. [28]).

The mathematical model enables the deduction of valuable information from the experimental results. First, from Eq. (14), the new, elevated, 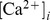 equilbrium reached after Rya-Caf treatment satisfies:
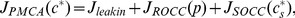
(13a)

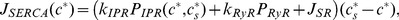
(13b)where 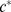 and 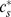 are respectively the equilibrium 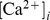 and 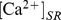. An important consequence of (18) is that, in the absence of SOCE, 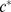 depends only on the 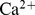 fluxes through the PM. This may seem surprising, as any increase in 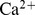 flux out of the SR (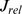 in Eq. (1)) is expected to increase 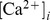. However, the equilibrium equation (18) tells us that such an increase would only be transient (because the PMCA pumping rate is an increasing function of 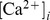 ), *unless* there is a concomitant permanent increase in 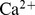 influx through the PM. Hence, the persistence of an elevated 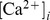 means that a permanent SOCE has been elicited (as SOCE is the only 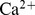 influx capable of increase upon Rya-Caf treatment). Moreover, the model indicates that ROCE is negligible after Rya-Caf treatment. Indeed, if it was not, the addition of agonist would increase 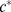 via the increase in 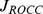. Hence, we assume that the ROCE rate 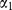 is small (see Table 1 and Discussion).

**Figure 4:**
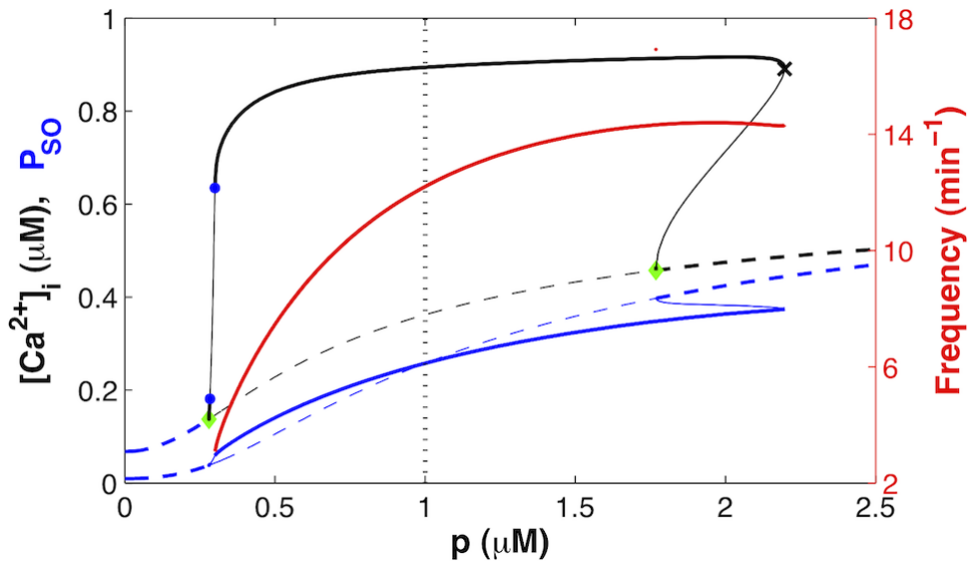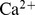 dynamics as a function of agonist concentration. Dashed curves represent steady-states (constant 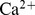 levels); solid curves, periodic solutions (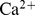 oscillations). The maximum 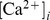 (black) and the maximum fraction of open SOCC (blue) during one solution period are plotted as ordinates. The red curve (right y-axis) shows the frequency of the 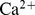 oscillations on the main stable segment (from the upper blue dot to the black cross), which fits the experimental range in human [2]. The stable solutions are represented as thick lines and unstable solutions as thin lines. The green diamonds represent Hopf bifurcations, the black cross, a saddle-node bifurcation, and the blue dots, period-doubling points. Period-doubled branches are not shown because they extend only over a tiny range of 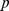 values; moreover it is likely that the deterministic description of 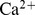 oscillations fails at these low agonist concentrations (see Discussion). The vertical dotted line indicates the value of 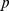 used in Fig. 3 (Table 1).

Results of “hand-fitting” the model to the experimental results are shown in Figs. 3D–F and Fig. 4, with the corresponding parameter values listed in Table 1. The model reproduces (i) the agonist-induced 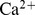 oscillations, (ii) the similar magnitudes of the new equilibrium 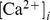 in Fig. 3B and the amplitude of the oscillations in Fig. 3A, and (iii) the negligible effect of agonist stimulation after Rya-Caf treatment. Agonist-induced 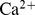 oscillations were simulated with 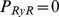 because RyR appear to play a negligible role during agonist-induced 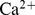 oscillations [2], [39]. On the other hand, the response to Rya-Caf was simulated with 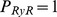 since the treatment locks open the RyR. We did not attempt to reproduce the magnitude of the initial spike response to Rya-Caf treatment relative to that of the subsequent 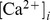 plateau (Fig. 3B) because the fluorescent dye used in the experiments saturates rapidly with 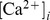. Parameter values were also adjusted to yield physiological 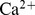 equilibrium concentrations (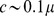
M [40] and 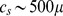
M [41]), realistic 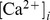 oscillation amplitude (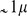
M), and to reproduce the range of 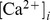 oscillation frequencies observed in human lung slices as a function of agonist (0.5–11/min [2]). More detail on the parameter estimation procedure is given in Supporting Information S1.

Fig. 4 shows the bifurcation diagram of the model as a function of agonist concentration. Periodic solutions (i.e., 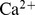 oscillations) arise through a Hopf bifurcation, and disappear through a saddle-node bifurcation of limit cycles. A second Hopf bifurcation is present on the steady-state branch, and is associated with a region of bistability between the steady-state and the periodic solution at the right of the bifurcation diagram. It is not known whether such bistability occurs in reality. It should also be noted that the steady-state 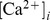 increases with agonist concentration, as is expected (e.g., [30]). This increase is provided by SOCE in our model. Indeed, the 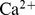 flux through IPR increases with agonist, so that store depletion increases as well.

### Effect of SOCE regulation on agonist-induced 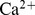 oscillations

**Figure 5:**
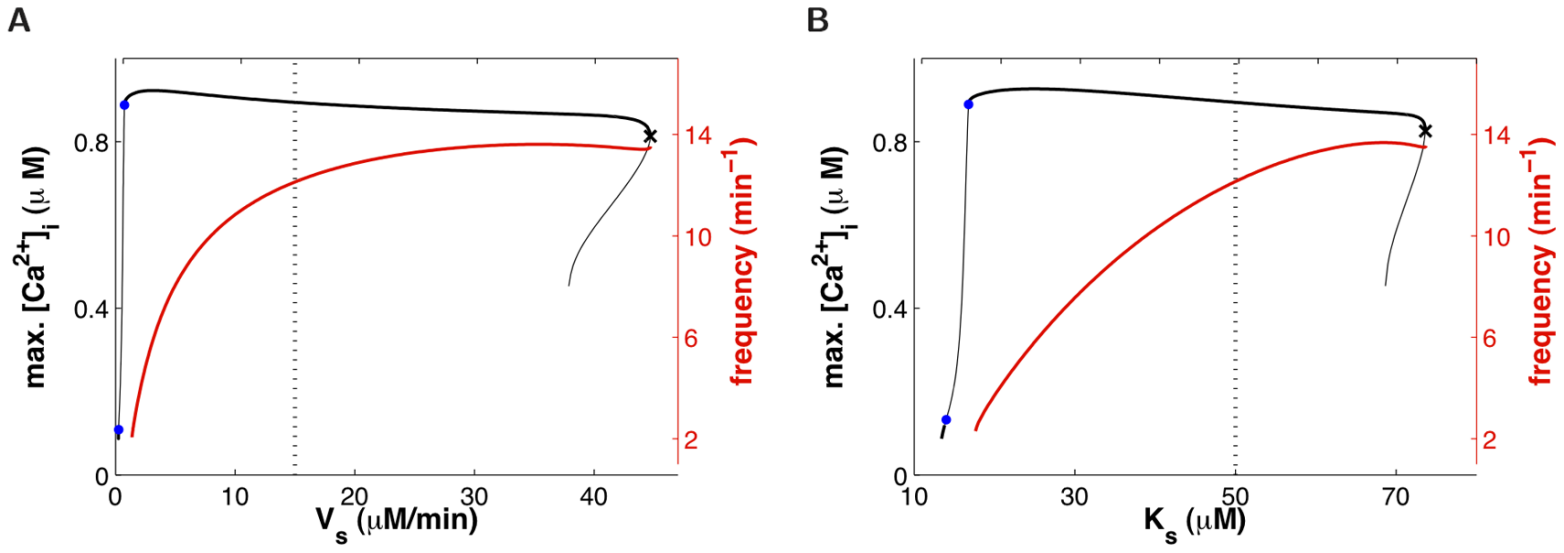
Influence of SOCE on agonist-induced 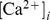
oscillations. Amplitude (black) and frequency (red) of 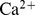 oscillations as a function of (A) SOCE maximum rate, 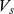, and (B) STIM affinity for SR 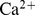, 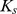. Dotted lines indicate the “normal” parameter values (Table 1, Figs. 3D–F). As in Fig. 4, only the frequency of the large-amplitude stable 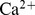 oscillations is shown.

SOCE is the main 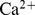 influx in the model, as ROCE is negligible (see above) and the 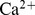 leak influx is (by definition) small. Fig. 3D shows that while SOCE is almost zero at physiological equilibrium (initial condition), it substantially increases during agonist-induced 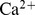 oscillations (final condition; see also Fig. 4), due to significant SR 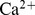 depletion. Therefore, changes in SOCE can be expected to have a substantial effect on 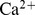 oscillations. This is quantified in Fig. 5, where the amplitude and frequency of 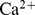 oscillations are plotted as a function of (a) the maximum SOCE rate, 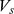, and (b) STIM affinity for 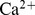, 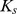 (the 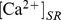 at which half SOCC are open). It is found that the 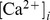 oscillation frequency varies as much with 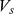 and 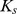 at fixed agonist concentration (Fig. 5) as it varies with agonist concentration at fixed SOCE parameters (Fig. 4). Moreover, a too big departure from the “normal” values (dotted lines, Table 1) leads to the extinction of the 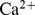 oscillations (via a Hopf bifurcation to the left, and a saddle-node to the right, of the bifurcation diagrams in Figs. 5A–B). These results are not very surprising to the extent that 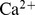 oscillations are expected to depend crucially on 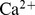 influx (e.g., [42]). However, they suggest that SOCE could play a role in AHR since (i) 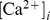 oscillations mediate ASMC contraction, and (ii) SOCE up-regulation (which increases 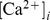 oscillation frequency) can be triggered by inflammatory mediators commonly found in asthma [9], [11], [24].

### Partial inhibition of SERCA by CPA

**Figure 6:**
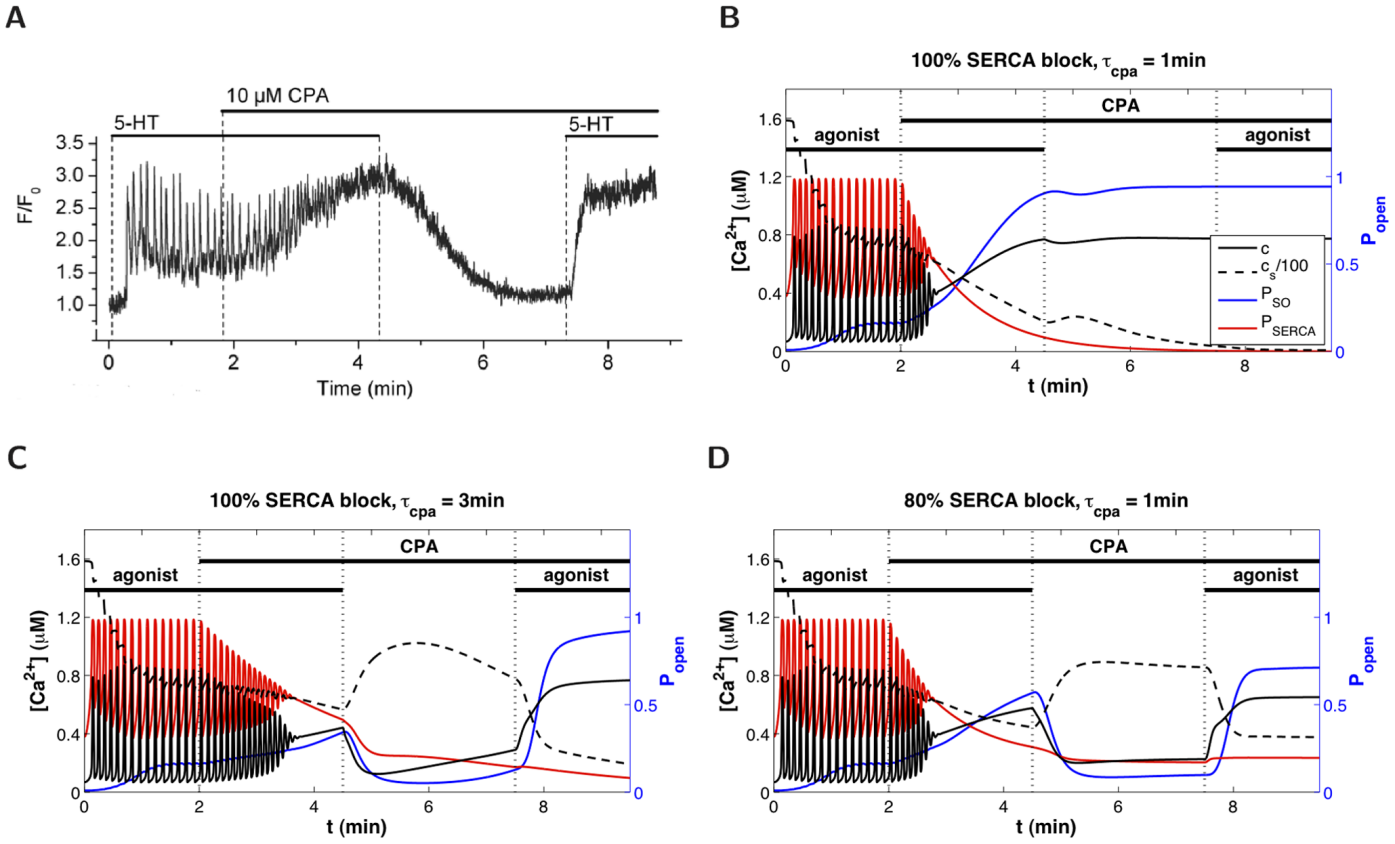
Effect of CPA on 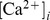 dynamics. (A) Fluorescence imaging of 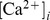 in ASMC of a mouse lung slice treated with agonist and CPA. Agonist removal leads to 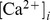 decrease. (B–D) Model simulations of the experiments shown in (A), assuming that (B) CPA quickly blocks the SERCA, (C) CPA slowly blocks the SERCA, (D) CPA partially blocks the SERCA but reaches maximum strength rather quickly. Black solid and dashed curves (left y-axis) represent respectively 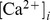 and 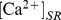; blue and red curves (right y-axis) show respectively the fraction of open SOCC and the fraction of operating SERCA (that is, 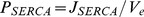, where 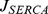 is given by Eq. (0c)).

We now apply the model to experimental data from mouse lung slices showing an attempt to clamp 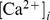 with the SERCA blocker CPA, instead of Rya-Caf treatment (Fig. 6). After inducing 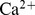 oscillations with agonist, CPA is applied in the presence of agonist (for faster emptying of the SR than CPA alone) and causes a gradual damping of the 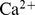 oscillations, together with a rise of the 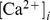 baseline, until the oscillations become undistinguishable from fluctuations around an elevated steady 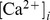 mean. Because CPA is believed to inhibit SERCA, the assumption, at this stage of the experiment, is that the SR is empty and SOCE fully active. However, when agonist is removed (CPA remains), 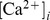 falls. When agonist is reapplied, 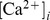 increases. These 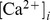 responses to agonist addition and removal are not observed when SOCE is evoked by Rya-Caf treatment. According to our model (Eq.(18)), the decrease in 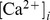 upon agonist removal indicates that SOCE does not remain activated, i.e. that the SR refills with 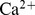. This suggests that the SERCA are not completely blocked by CPA, as illustrated by the simulations in Fig. 6B–D. If CPA was to fully block the SERCA (Fig. 6B), 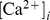 would not decrease upon agonist removal. If 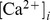 falls, it must be because either CPA requires a longer time than that used in the experiment to fully block the SERCA (Fig. 6C), or CPA achieves only partial block of the SERCA (Fig. 6D).

**Figure 7:**
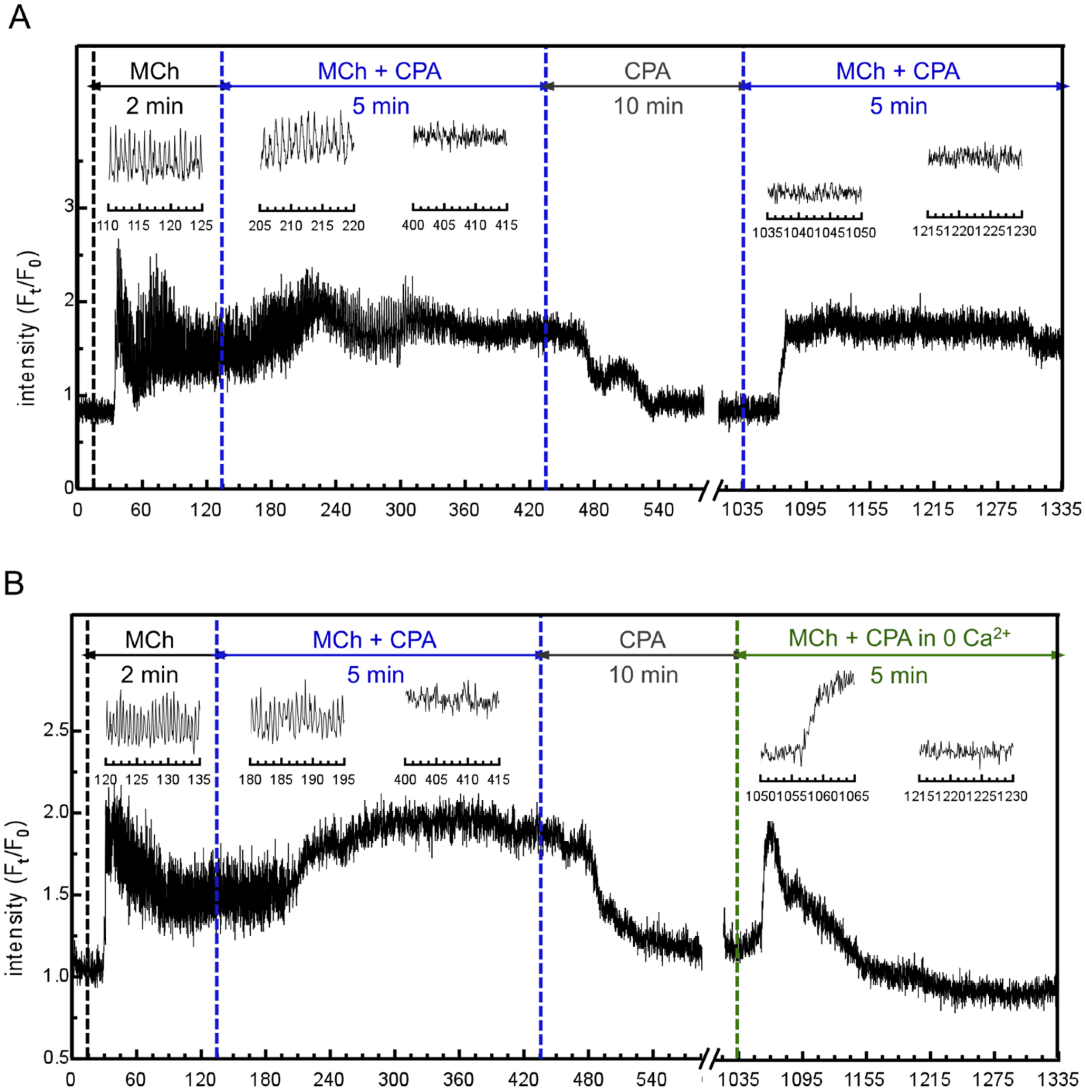
Experimental evidence that CPA does not fully empty the SR of ASMC. Tests of the model predictions shown in Fig. 6B–D, performed with mouse lung slices. (A) Significantly longer exposure to agonist+CPA and to CPA than in Fig. 6A still fails to maintain SOCE. (B) Same experiment as in (A) except that extracellular 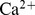 is removed before agonist is applied a second time, confirming the residual presence of 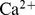 in the SR and hence the partial efficacy of CPA to inhibit SERCA (scenario of Fig. 6D). (Insets show magnifications of selected time windows).

Experiments of longer duration were performed to test the model predictions. Fig. 7A shows that if CPA is applied in the presence of agonist for 5 minutes, followed by CPA only for a further 10 minutes, 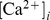 still returns to the original equilibrium level when agonist is removed, and remains low until agonist is reintroduced. This suggests that the explanation in Fig. 6C can be rejected, otherwise the longer exposure to CPA should yield a result similar to Fig. 6B. The inability of CPA to fully empty the SR of 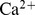 is confirmed by Fig. 7B, where extracellular calcium is removed before agonist is applied a second time, to prevent any potential ROCE. The 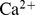 response induced can thus be unambiguously attributed to 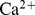 release from the SR.

Hence, our combined modelling and experimental study indicates that CPA blocks only partially the SERCA of ASMC in lung slices (scenario simulated in Fig. 6D). This is a potentially important result given the wide use of CPA in cell biology to study SOCE. We note that Figs. 6A and 7 could also be explained by a model assuming that ROCE, instead of SOCE, is the main 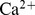 influx (e.g., [23]). However, such a model would fail to explain the outcome of Rya-Caf treatment in human and mouse lung slices (both the persistent elevated 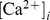 in the absence of agonist, and the absence of effect of agonist on this elevated 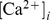 ). In contrast, our model, constructed to account for both agonist-induced oscillations and Rya-Caf treatment, explains the CPA results without requiring any modification. Its prediction holds provided CPA is not a 100% efficient SERCA blocker, and this hypothesis is supported by the experimental data in Fig. 7.

## Discussion

### Modelling SOCE

Our mathematical model accounts for the two main properties of SOCE: 1) SOCE is an increasing function of 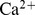 store depletion, and 2) it activates slowly upon store depletion. While the mechanisms of SOCE activation are rather well understood [14], [32], the mechanisms of SOCE termination remain less clear [43], [44]. Hence, we do not explicitly distinguish between SOCE activation and inactivation in the model, and use a single parameter 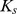 for STIM affinity for SR 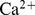 and a single time constant 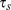 for the slow adaptation to changes in 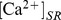. This is also justified by the fact that most experimental data available on SOCE come from a category of SOCC called CRACC (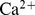 -release-activated 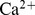 channels), which are highly selective to 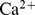, while there is evidence that SOCE in ASMC (and in other cells) occurs at least in part through non-selective 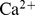 channels (NSCC). It could be that the latter operate somewhat differently from CRACC in response to store depletion or refilling.

Our description of SOCE slow activation upon store depletion is continuous, which is easy to handle computationally, and compatible with experimental knowledge. Indeed, it is reasonable to assume that a small fraction of STIM proteins reside in close proximity to the PM, and may thus bind Orai quickly upon store depletion. Hence, a weak SOCE is likely to occur almost instantaneously upon store depletion, rendering unnecessary to introduce a finite activation delay in the model via a delay-differential equation.

We are aware of only few prior works on 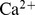 dynamics that include a mathematical description of SOCE, all of which are ODE models [15]–[18]. The first two were published before the molecular basis for SOCE was established. The latter two works include more realistic descriptions of SOCE, but none of them accounts for the slow translocation of oligomerised STIM to the PM, while it is recognised as the rate-limiting event for SOCE activation [32]. Ong *et al*. however assume a slow diffusion of 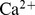 between internal SR and superficial SR (modelled as distinct compartments exchanging 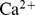 ), with SOCE being triggered by peripheral SR depletion [17]. Liu *et al*. explicitly model both SR 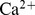 dissociation from STIM and binding of STIM to Orai. Both models are used to study transient 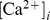 responses only; 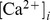 oscillations are not considered. Prior models of 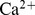 dynamics specific to ASMC did not include SOCE, while we have shown that this is necessary to account for several experimental results obtained with lung slices. The work of Haberichter *et al*. [19] focused on the influence of the different IPR isoforms on 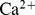 signalling in ASMC. Brumen *et al*. studied the influence of the total 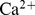 content on the nature (damped or sustained) and frequency of agonist-induced 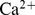 oscillations [21]. Roux *et al.* did not model 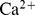 oscillations, but transient 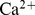 responses to caffeine [20]. Finally, the model by Wang *et al.* [23] addressed the different contributions of IPR and RyR to agonist-induced and KCl-induced 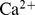 oscillations in ASMC.

From the mathematical point of view, the fact that SOCE is an explicit function of store 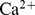 renders the models of 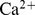 dynamics including this influx qualitatively different from those which do not, as SOCE couples the homogenous steady-state 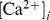 to 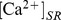 (Eq. (18)). This property is essential for the predictions of our model (in particular, the persistence of an elevated 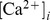 upon sustained store depletion in the absence of agonist). On the other hand, whether SOCE is an instantaneous or delayed function of 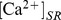 appears to have little effect on our results.

### SOCE vs. ROCE

While Fig. 3C (as well as Fig. 6 in ref. [29], Figs. 5B and 6C-D in ref. [28]) shows that no ROCE is elicited by agonist following Rya-Caf treatment, it does not imply that ROCE cannot play a substantial role during other, more physiological, conditions, such as agonist-induced 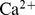 oscillations. It could be that ROCE is inhibited at the large 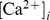 levels induced by SOCE activation following Rya-Caf treatment. Instead of assuming the existence of an inactivation process at large 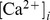, we assumed, for simplicity, that ROCE is negligible in the model. This approach enabled us to show that 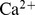 influx through SOCC is sufficient to sustain agonist-induced 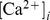 oscillations, and to explain the experimental results obtained with CPA, although the latter could be interpreted as evidence for ROCE at first sight. The fact that there appears to be no selective blocker for SOCE and ROCE makes it difficult to evaluate experimentally the respective contributions of the two 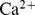 influxes during physiological conditions. These magnitudes are probably also cell-type dependent. Such issues explain the persistence of the controversy regarding SOCE and ROCE [45]–[48]. An informative experiment would be to stimulate ASMC using flash photolysis of caged 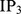 instead of agonist stimulation. Indeed, as 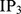 does not induce ROCE, SOCE should be the essential 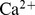 influx left. By comparing the responses to 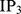 stimulation in the presence and in the absence of extracellular calcium, one could then deduce the importance of SOCE in physiological conditions.

### Efficacy of CPA

CPA is widely used as a SERCA blocker, having the advantage over Thapsigargin (Tg) of being reversible, and probably less toxic. Both have been used extensively to study SOCE in different cell types (e.g., [25]–[27], [43], [49]). Although our work indicates that CPA does not fully block the SERCA in intact tissue such as lung slices, it does not imply that CPA should not be used experimentally to induce SOCE. Indeed, CPA might still cause substantial SOCE activation in the presence of agonist. However, our results indicate that CPA is not a good mean to fully empty 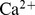 stores, and care should be taken in interpreting the experimental results of its application. We suggest that a combined Rya-Caf treatment is a more reliable way to induce a permanent large SR depletion (Fig. 3B, C). There is evidence that Tg is an efficient SERCA blocker in cell lines such as Hela cells [43], but we have not addressed the effect of Tg on ASMC in lung slices in this study.

### Modelling IPR

In this work, we followed the approach of Wang *et al.* [23], in that we have used one of the simplest models of IPR 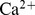 release, namely the Li-Rinzel/Tang *et al.* reduction of the DYK ODE model [34]–[36]. This category of IPR model produces agonist-induced 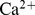 oscillations characterised by significant SR 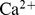 depletion (Fig. 3D and [23]), hence the possibility of SOCE being activated during such 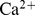 oscillations. This property might be model-dependent, however there is evidence that the SR is actually depleted to some extent during agonist-induced 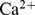 oscillations in ASMC. Indeed, the absence of effect of ryanodine during agonist-induced oscillations can be explained by the average level of 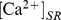 being too low for RyR activation [1], [23]. However, the respective 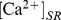 “thresholds” for SOCE and RyR activation are experimentally unknown. In this work, the SOCE activation threshold was deduced from fitting the model simultaneously to Fig. 3A and Figs. 3B–C.

Finally, we note that our whole-cell 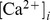 model would likely not benefit from using a recent Markov model of an IPR (e.g., [50]–[52]), because these models are based on steady-state data only (i.e., single-channel opening and closing times in stationary 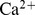 and 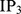) and typically miss the long inactivation timescale which was included “ad hoc” in the first IPR models to reproduce the observed behavior at the cell level (i.e., 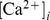 oscillations upon agonist stimulation).

### Limitations of the whole-cell model

As we are essentially interested in 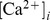 responses of ASMC at the cell level, we have described 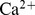 dynamics via a deterministic ODE model. The scope of this model is, however, somewhat limited for the following reasons.

First, there is evidence that IPR are not homogeneously distributed on the SR membrane of cells, but are found as dense clusters. This channel clustering is especially patent upon stimulation by low agonist concentrations, for which local, stochastic 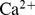 releases may not propagate to neighboring clusters, resulting in spatially isolated, unsynchronised 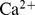 releases, called “puffs”. At higher agonist concentrations, the frequency of these puffs increases, allowing 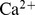 releases from close sites to accumulate and propagate further away. This triggers, via CICR, the firing of more distant clusters, and results in 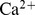 waves propagating repeatedly throughout the cytosol. These waves usually appear as 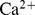 oscillations at the whole-cell level. While 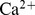 waves are indeed associated with 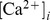 oscillations in ASMC [1], it has, so far, been impossible to detect 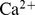 puffs. This could arise from a less clustered distribution of IPR in ASMC, compared to the larger cells (ooycytes and Hela cells) where puffs have been characterised. On the other hand, 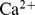 “sparks”, the equivalent of 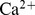 puffs but mediated by RyR, have been detected in ASMC [1], which supports a clustered distribution of RyR. In this study, we did not attempt to consider these spatial/stochastic aspects of the 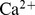 signals. Our model is thus less reliable at low agonist concentrations.

Second, cytoplasmic microdomains often exist between cell organelles (e.g., between peripheral SR and the plasma membrane, between the SR and mitochondria), out of which 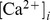 cannot diffuse easily. These have consequences for SOCE dynamics. Indeed, it has been reported that upon store depletion, SERCA can colocalise with STIM proteins, in proximity to the PM [49], [53]. As a consequence, if SOCE is slow enough, the SR can refill with 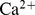 without a concomitant increase in bulk 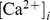 [49]. Upon large SOCE, this is no longer the case; however, mitochondria prevent the local 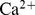 increase to become too large by pumping 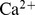 from the subplasmalemmal space and releasing it deeper in the cytoplasm, where it can be absorbed by other SERCA [49]. These spatial effects cannot be accounted for by our current non-compartmentalised model.

Finally, 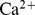 dynamics are modified by 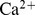 buffers in the cytosol and SR, which bind 99% of the free 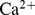. While the effect of fast, linear buffers can be taken into account by a global rescaling of 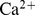 fluxes (see Methods), this is not the case for high affinity buffers, in particular fluorescent dye indicators. Including such buffers in an ODE model of 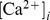 dynamics leads to suppression of 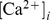 oscillations, because the buffer affinity is close to the amplitude of whole-cell 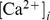 oscillations. In reality, 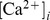 reaches much higher levels locally upon IPR opening, so that the buffers become saturated and cannot prevent 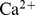 oscillations. Again, this would have to be accounted for by a spatial model of 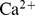 dynamics.

### Future work

Although RyR dynamics play a role only during the initial phases of agonist-induced 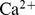 oscillations and Rya-Caf treatment, the interaction between RyR and IPR may become important in other situations, such as drug-induced RyR sensitisation. We plan to extend our model to these dynamics.

Since our work is part of a broader effort to improve the understanding of airway hyper-responsiveness and remodelling via mathematical modelling [54]–[57], we also intent to model the interaction of ASMC 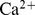 signalling with other aspects of lung dynamics. Although mathematical models of ASM contraction have previously been developed [54], [55], [58], modelling of other signalling pathways, such as inflammation and proliferation, is, to our knowledge, still in its infancy.

Additionally, experimental studies of ASMC inflammation and proliferation in conjunction with 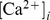 imaging in lung slices would be desirable. While such studies have been carried out with cultured ASMC [3]–[5], [9]–[12], they do not provide individual 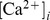 dynamics; moreover, cultured ASMC often exhibit a different phenotype from ASMC in intact tissues.

## Conclusions

The inclusion of SOCE in our mathematical model of 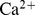 dynamics in ASMC enables a better understanding of the experimental physiology of lung slices. It shows that the different abilities of CPA and Rya-Caf treatment to clamp the 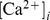 of ASMC can be explained by their different ability to invoke SOCE. The model predicts that CPA, in contrast with Rya-Caf treatment, is unable to empty the SR because of its inefficiency to fully inhibit the SERCA. Furthermore, by accounting for both agonist-induced 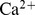 oscillations and SOCE activation by SR 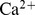 depletion, the model shows that SOCE can be a major determinant of the frequency of agonist-induced 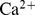 oscillations. Because this frequency of the 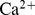 oscillations regulates airway contraction, the model suggests a role for increased SOCE in AHR, a correlation consistent with SOCE up-regulation under inflammatory conditions typical of asthma. These predictions underscore the synergistic role for mathematical modeling in medical research.

## Supporting Information

Supporting Information S1 **Details of the parameter estimation procedure.**
(PDF)

## References

[1] Perez JF, Sanderson MJ (2005) The frequency of calcium oscillations induced by 5-HT, ACH, and KCl determine the contraction of smooth muscle cells of intrapulmonary bronchioles. J Gen Physiol125: 535–53. 10.1085/jgp.200409216 PMC2234076 15928401

[2] Ressmeyer AR, Bai Y, Delmotte PF, Uy KF, Thistlethwaite P, et al (2010) Human airway contraction and formoterol-induced relaxation is determined by Ca^2+^ oscillations and Ca^2+^ sensitivity. Am J Respir Cell Mol Biol 43: 179–91. 19767449 10.1165/rcmb.2009-0222OC PMC2937231

[3] Sweeney M, McDaniel SS, Platoshyn O, Zhang S, Yu Y, et al (2002) Role of capacitative Ca^2+^ entry in bronchial contraction and remodeling. J Appl Physiol 92: 1594–602. 11896026 10.1152/japplphysiol.00722.2001

[4] Mahn K, Hirst SJ, Ying S, Holt MR, Lavender P, et al (2009) Diminished sarco/endoplasmic reticulum Ca^2+^ ATPase (SERCA) expression contributes to airway remodelling in bronchial asthma. PNAS 106: 10775–80. 19541629 10.1073/pnas.0902295106 PMC2699374

[5] Zou JJ, Gao YD, Geng S, Yang J (2011) Role of STIM1/Orai1-mediated store-operated Ca^2+^ entry in airway smooth muscle cell proliferation. J Appl Physiol 110: 1256–63. 21330611 10.1152/japplphysiol.01124.2010

[6] Gerthoffer WT (1991) Regulation of the contractile element of airway smooth muscle. Am J Physiol Lung Cell Mol Physiol 5: L15–L28. 10.1152/ajplung.1991.261.2.L15 1872409

[7] Janmey PA (1994) Phosphoinositides and calcium as regulators of cellular actin assembly and disassembly. Annu Rev Physiol 56: 169–91. 8010739 10.1146/annurev.ph.56.030194.001125

[8] Herrera AM, Kuo KH, Seow CY (2002) Influence of calcium on myosin thick filament formation in intact airway smooth muscle. Am J Physiol Cell Physiol 282: C310–6. 11788342 10.1152/ajpcell.00390.2001

[9] White TA, Xue A, Chini EN, Thompson M, Sieck GC, et al (2006) Role of transient receptor potential C3 in TNF-alpha-enhanced calcium influx in human airway myocytes. Am J Respir Cell Mol Biol 35: 243–51. 16574942 10.1165/rcmb.2006-0003OC PMC2643259

[10] Moynihan B, Tolloczko B, Michoud MC, Tamaoka M, Ferraro P, et al (2008) MAP kinases mediate interleukin-13 effects on calcium signaling in human airway smooth muscle cells. Am J Physiol Lung Cell Mol Physiol 295: L171–7. 18441092 10.1152/ajplung.00457.2007 PMC2494781

[11] Sieck GC, White TA, Thompson MA, Pabelick CM, Wylam ME, et al (2008) Regulation of store-operated Ca^2+^ entry by CD38 in human airway smooth muscle. Am J Physiol Lung Cell Mol Physiol 294: L378–85. 18178673 10.1152/ajplung.00394.2007

[12] Sathish V, Thompson MA, Bailey JP, Pabelick CM, Prakash YS, et al (2009) Effect of proinflammatory cytokines on regulation of sarcoplasmic reticulum Ca^2+^ reuptake in human airway smooth muscle. Am J Physiol Lung Cell Mol Physiol 297: L26–34. 19395670 10.1152/ajplung.00026.2009 PMC2711800

[13] Putney JW (1986) A model for receptor-regulated calcium entry. Cell Calcium 7: 1–12. 2420465 10.1016/0143-4160(86)90026-6

[14] Roos J, DiGregorio PJ, Yeromin AV, Ohlsen K, Lioudyno M, et al (2005) STIM1, an essential and conserved component of store-operated Ca^2+^ channel function. J Cell Biol 169: 435–45. 15866891 10.1083/jcb.200502019 PMC2171946

[15] Wiesner TF, Berk BC, Nerem RM (1996) A mathematical model of cytosolic calcium dynamics in human umbilical vein endothelial cells. Am J Physiol Cell Physiol 270: C1556–C1569. 10.1152/ajpcell.1996.270.5.C1556 8967458

[16] Kowalewski JM, Uhlén P, Kitano H, Brismar H (2006) Modeling the impact of store-operated Ca^2+^ entry on intracellular Ca^2+^ oscillations. Math Biosci 204: 232–49. 16620876 10.1016/j.mbs.2006.03.001

[17] Ong HL, Liu X, Tsaneva-Atanasova K, Singh BB, Bandyopadhyay BC, et al (2007) Relocalization of STIM1 for activation of store-operated Ca^2+^ entry is determined by the depletion of subplasma membrane endoplasmic reticulum Ca^2+^ store. J Biol Chem 282: 12176–85. 17298947 10.1074/jbc.M609435200 PMC3309416

[18] Liu W, Tang F, Chen J (2010) Designing Dynamical Output Feedback Controllers for Store-operated Ca^2+^ Entry. Math Biosci 228: 110–118. 20816868 10.1016/j.mbs.2010.08.013

[19] Haberichter T, Roux E, Marhl M, Mazat JP (2002) The influence of different InsP3 receptor isoforms on Ca^2+^ signaling in tracheal smooth muscle cells. Bioelectrochemistry 57: 129–38. 12160609 10.1016/s1567-5394(02)00063-4

[20] Roux E, Marhl M (2004) Role of sarcoplasmic reticulum and mitochondria in Ca^2+^ removal in airway myocytes. Biophys J 86: 2583–95. 15041694 10.1016/S0006-3495(04)74313-1 PMC1304105

[21] Brumen M, Fajmut A, Dobovišek A, Roux E (2005) Mathematical Modelling of Ca^2+^ Oscillations in Airway Smooth Muscle Cells. J Biol Phys 31: 515–524. 23345915 10.1007/s10867-005-2409-4 PMC3456323

[22] Roux E, Noble PJ, Noble D, Marhl M (2006) Modelling of calcium handling in airway myocytes. Prog Biophys Mol Biol 90: 64–87. 15982722 10.1016/j.pbiomolbio.2005.05.004

[23] Wang IY, Bai Y, Sanderson MJ, Sneyd J (2010) A mathematical analysis of agonist- and KCl- induced Ca^2+^ oscillations in mouse airway smooth muscle cells. Biophys J 98: 1170–81. 20371316 10.1016/j.bpj.2009.12.4273 PMC2849087

[24] Gao YD, Zou JJ, Zheng JW, Shang M, Chen X, et al (2010) Promoting effects of IL-13 on Ca^2+^ release and store-operated Ca^2+^ entry in airway smooth muscle cells. Pulm Pharmacol Ther 23: 182–9. 20045483 10.1016/j.pupt.2009.12.005

[25] Ay B, Prakash YS, Pabelick CM, Sieck GC (2004) Store-operated Ca^2+^ entry in porcine airway smooth muscle. Am J Physiol Lung Cell Mol Physiol 286: L909–17. 14617522 10.1152/ajplung.00317.2003

[26] Peel SE, Liu B, Hall IP (2006) A key role for STIM1 in store-operated calcium channel activation in airway smooth muscle. Respir Res 7: 119. 16987424 10.1186/1465-9921-7-119 PMC1584236

[27] Peel SE, Liu B, Hall IP (2008) ORAI and store-operated calcium inux in human airway smooth muscle cells. Am J Respir Cell Mol Biol 38: 744–9. 18239188 10.1165/rcmb.2007-0395OC PMC2643203

[28] Bai Y, Sanderson MJ (2006) Modulation of the Ca^2+^ sensitivity of airway smooth muscle cells in murine lung slices. Am J Physiol Lung Cell Mol Physiol 291: L208–21. 16461427 10.1152/ajplung.00494.2005

[29] Bai Y, Sanderson MJ (2009) The contribution of Ca^2+^ signaling and Ca^2+^ sensitivity to the regulation of airway smooth muscle contraction is different in rats and mice. Am J Physiol Lung Cell Mol Physiol 296: L947–58. 19346434 10.1152/ajplung.90288.2008 PMC2692797

[30] Keener J, Sneyd J (2008) Mathematical physiology, second edition. Springer.

[31] Janssen LJ (2002) Ionic mechanisms and Ca^2+^ regulation in airway smooth muscle contraction: do the data contradict dogma? Am J Physiol Lung Cell Mol Physiol 282: L1161–78. 12003770 10.1152/ajplung.00452.2001

[32] Liou J, Fivaz M, Inoue T, Meyer T (2007) Live-cell imaging reveals sequential oligomerization and local plasma membrane targeting of stromal interaction molecule 1 after Ca^2+^ store depletion. PNAS 104: 9301–6. 17517596 10.1073/pnas.0702866104 PMC1890489

[33] Luik R, Wang B, Prakriya M, Wu M, Lewis R (2008) Oligomerization of STIM1 couples ER calcium depletion to CRAC channel activation. Nature 454: 538–542. 18596693 10.1038/nature07065 PMC2712442

[34] De Young GW, Keizer J (1992) A single-pool inositol 1, 4, 5-trisphosphate-receptor-based model for agonist-stimulated oscillations in Ca^2+^ concentration. PNAS 89: 9895–9899. 1329108 10.1073/pnas.89.20.9895 PMC50240

[35] Li Y, Rinzel J (1994) Equations for InsP3 receptor-mediated [Ca^2+^]i oscillations derived from a detailed kinetic model: a Hodgkin-Huxley like formalism. J Theor Biol 166: 461–473. 8176949 10.1006/jtbi.1994.1041

[36] Tang Y, Stephenson JL, Othmer HG (1996) Simplification and analysis of models of calcium dynamics based on IP3-sensitive calcium channel kinetics. Biophys J 70: 246–63. 8770202 10.1016/S0006-3495(96)79567-X PMC1224924

[37] Doedel EJ, Keller HB, Kernevez JP (1991) Numerical analysis and control of bifurcation problems: (I) Bifurcation in finite dimensions. Int J Bifurc Chaos 1: 493–520.

[38] Doedel EJ, Keller HB, Kernevez JP (1991) Numerical analysis and control of bifurcation problems: (II) Bifurcation in infinite dimensions. Int J Bifurc Chaos 1: 745–772.

[39] Bai Y, Edelmann M, Sanderson MJ (2009) The contribution of inositol 1,4,5-trisphosphate and ryanodine receptors to agonist-induced Ca^2+^ signaling of airway smooth muscle cells. Am J Physiol Lung Cell Mol Physiol 297: L347–61. 19465516 10.1152/ajplung.90559.2008 PMC2742787

[40] Berridge MJ (2009) Cell Signalling Pathways. In: Cell Signalling Biology, Portland Press, volume 3: 2.1–2.118 Available: www.cellsignallingbiology.org.

[41] Demaurex N, Frieden M (2003) Measurements of the free luminal ER Ca^2+^ concentration with targeted cameleon fluorescent proteins. Cell Calcium 34: 109–119. 12810053 10.1016/s0143-4160(03)00081-2

[42] Sneyd J, Tsaneva-Atanasova K, Yule DI, Thompson JL, Shuttleworth TJ (2004) Control of calcium oscillations by membrane fluxes. PNAS 101: 1392–6. 14734814 10.1073/pnas.0303472101 PMC337063

[43] Shen WW, Frieden M, Demaurex N (2011) Local cytosolic Ca^2+^ elevations are required for stromal interaction molecule 1 (STIM1) de-oligomerization and termination of store-operated Ca^2+^ entry. J Biol Chem 286: 36448–59. 21880734 10.1074/jbc.M111.269415 PMC3196111

[44] Malli R, Naghdi S, Romanin C, Graier WF (2008) Cytosolic Ca^2+^ prevents the subplasmalemmal clustering of STIM1: an intrinsic mechanism to avoid Ca^2+^ overload. J Cell Sci 121: 3133–9. 18765567 10.1242/jcs.034496 PMC4064434

[45] Shuttleworth TJ (1999) What drives calcium entry during [Ca^2+^]i oscillations? – challenging the capacitative model. Cell Calcium 25: 237–46. 10378085 10.1054/ceca.1999.0022

[46] Bird GSJ, Putney JW (2005) Capacitative calcium entry supports calcium oscillations in human embryonic kidney cells. J Physiol 562: 697–706. 15513935 10.1113/jphysiol.2004.077289 PMC1665541

[47] Trebak M (2011) PLC: Johnny-come-lately to ORAI and the ups and downs of calcium signalling. J Physiol 589: 5337–8. 22086246 10.1113/jphysiol.2011.220517 PMC3240871

[48] Shuttleworth T (2012) STIM and Orai proteins and the non-capacitative ARC channels. Front Biosci 17: 847–860. 10.2741/3960 PMC3295580 22201777

[49] Jousset H, Frieden M, Demaurex N (2007) STIM1 knockdown reveals that store-operated Ca^2+^ channels located close to sarco/endoplasmic Ca^2+^ ATPases (SERCA) pumps silently refill the endoplasmic reticulum. J Biol Chem 282: 11456–64. 17283081 10.1074/jbc.M609551200

[50] Mak DOD, McBride SMJ, Foskett JK (2003) Spontaneous channel activity of the inositol 1,4,5- trisphosphate (InsP3) receptor (InsP3R). Application of allosteric modeling to calcium and InsP3 regulation of InsP3R single-channel gating. J Gen Physiol 122: 583–603. 14581584 10.1085/jgp.200308809 PMC2229577

[51] Gin E, Wagner LE, Yule DI, Sneyd J (2009) Inositol trisphosphate receptor and ion channel models based on single-channel data. Chaos 19: 037104. 19792029 10.1063/1.3184540 PMC5848693

[52] Siekmann I, Wagner LE, Yule D, Crampin EJ, Sneyd J (2012) A Kinetic Model for type I and II IP3R accounting for mode changes. Biophys J 103: 658–68. 22947927 10.1016/j.bpj.2012.07.016 PMC3443778

[53] Manjarrés IM, Rodríguez-García A, Alonso MT, García-Sancho J (2010) The sarco/endoplasmic reticulum Ca^2+^ ATPase (SERCA) is the third element in capacitative calcium entry. Cell Calcium 47: 412–8. 20347143 10.1016/j.ceca.2010.03.001

[54] Wang I, Politi AZ, Tania N, Bai Y, Sanderson MJ, et al (2008) A mathematical model of airway and pulmonary arteriole smooth muscle. Biophys J 94: 2053–64. 18065464 10.1529/biophysj.107.113977 PMC2257911

[55] Brook BS, Peel SE, Hall IP, Politi AZ, Sneyd J, et al (2010) A biomechanical model of agonist-initiated contraction in the asthmatic airway. Respir Physiol Neurobiol 170: 44–58. 19932770 10.1016/j.resp.2009.11.006

[56] Politi AZ, Donovan GM, Tawhai MH, Sanderson MJ, Lauzon AM, et al (2010) A multiscale, spatially distributed model of asthmatic airway hyper-responsiveness. J Theor Biol 266: 614–624. 20678506 10.1016/j.jtbi.2010.07.032 PMC2939299

[57] Lauzon AM, Bates JHT, Donovan G, Tawhai M, Sneyd J, et al (2012) A multi-scale approach to airway hyperresponsiveness: from molecule to organ. Front Physiol 3: 191 (1–25).. 22701430 10.3389/fphys.2012.00191 PMC3371674

[58] Brook BS, Jensen OE (2013) The role of contractile unit reorganization in force generation in airway smooth muscle. Math Med Biol. In press. 10.1093/imammb/dqs031 PMC4453871 23360777

[59] O'Donnell ME, Owen NE (1994) Regulation of ion pumps and carriers in vascular smooth muscle. Physiol Rev 74: 683–721. 8036250 10.1152/physrev.1994.74.3.683

[60] Alberts B, Johnson A, Lewis J, Raff M, Roberts K, et al. (2008) Molecular Biology of the cell. New York: Garland, fifth edition. Available: http://www.worldcat.org/isbn/0815332181.

[61] Lytton J, Westlin M, Burk SE, Shull GE, MacLennan DH (1992) Functional comparisons between isoforms of the sarcoplasmic or endoplasmic reticulum family of calcium pumps. J Biol Chem 267: 14483–9. 1385815

