# Supplementary material for: Activation of Store-Operated Calcium Entry in Airway Smooth Muscle Cells: Insight from a Mathematical Model
Source: PLoS One. 2013 Jul 25;8(7):e69598. doi: 10.1371/journal.pone.0069598 (PMC3723852; doi:10.1371/journal.pone.0069598)
Supplement: Supporting Information S1 — Details of the parameter estimation procedure. (PDF) [file pone.0069598.s001.pdf]

Supporting Information S1 to:

"Activation of store-operated calcium entry in airway smooth muscle cells: insight from a mathematical model"

Huguette Croisier[1,*], Xiahui Tan[2], Jose F. Perez-Zoghbi[3], Michael J. Sanderson[2], James Sneyd[4], Bindi S. Brook[1]

1 School of Mathematical Sciences, University of Nottingham, Nottingham, UK.
2 Department of Microbiology and Physiological Systems, University of Massachusetts Medical School, Worcester, MA, USA.
3 Department of Cell Physiology and Molecular Biophysics, Texas Tech University Health Sciences Center, Lubbock, TX, USA.
4 Department of Mathematics, University of Auckland, Auckland, New Zealand.
∗ E-mail: Huguette.Croisier@nottingham.ac.uk

Parameter estimation procedure

In this section, we give more detail about the rationale which has guided our parameter estimation. From Eq. (13b), in the absence of agonist, the equilibrium Ca^{2+} concentrations obey:

$$c_s^* = c^* + \frac{V_e}{J_{SR}} \frac{c^{*2}}{K_e^2 + c^{*2}}.$$

Assuming $c^* \sim K_e$ (that is, assuming $K_e \sim 0.1\mu$M), to satisfy the experimental constraint $c_s^*/c^* \gg 1$, we must have $\frac{J_{SR}}{V_e/K_e} \ll 1$. Also, as J_{leakSR} represents a small Ca^{2+} leak through the SR membrane, it must be that $\frac{J_{SR}}{k_{IPR}} \ll 1$. These are our first two constraints on parameter values.

We must also have $\frac{k_{RyR}}{J_{SR}} \gg 1$ for the SR to be largely depleted by the Rya-Caf treatment, so that (i) SOCE is activated ($c_s \lesssim K_s$) and (ii) agonist stimulation is no longer able to trigger substantial Ca^{2+} release from the SR after the treatment.

The IPR rate k_{IPR} must also be large enough so that for $p/K_1 \gg 1$ (for which P_{IPR} is saturated as a function of p, cf. Eq. (7)), Ca^{2+} oscillations cease to exist. Otherwise, stable Ca^{2+} oscillations persist in an unphysiologically large, and possibly semi-infinite, range of agonist concentrations (this can happen if the ROCE rate is zero, because then, for $p/K_3 \gg 1$, increasing p no longer influences Ca^{2+} dynamics, as Φ_1 and Φ_2 are saturated (Eq. (9)).

Moreover, in order to have $c_s^* > 100\mu$M (to match the experimental estimation $c_s^* \sim 500\mu$M [1]), which is equivalent to $c_s^* > 1000c^*$, the quantity $\frac{V_e/K_e}{J_{SR}}$ must be so large that to keep the $[Ca^{2+}]_i$oscillation amplitude reasonable, we have to set simultaneously the maximum PMCA rate V_p to $V_p \sim V_e$ and the PMCA affinity to $K_p \sim 1\mu$M. Increasing only V_p kills the oscillations, and changing k_{IPR} has little effect on the oscillation amplitude. At the same time, SOCE must be large enough compared with the PMCA Ca^{2+} flux to maintain an elevated $[Ca^{2+}]_i$after the Rya-Caf treatment. As a consequence, the Ca^{2+} fluxes through the PM are not negligible in our model, and the total Ca^{2+} concentration $c_t = c + c_s/\gamma$ cannot be considered as a slow variable for model reduction purpose, as it is in several models of Ca^{2+} dynamics (e.g., [2–4]). In order to make that approximation, we would need $c_s \lesssim 100\,c$, but this would disagree with the current experimental estimations.

Finally, it is instructive to consider non-dimensional estimates of the relative magnitudes of the different Ca^{2+} fluxes in the model, since these ratios control the qualitative dynamics of Ca^{2+}. This is done in Table S1, together with a comparison with the same quantities in the Wang *et al.* model [4]. We note, in particular, that the ratio between the PMCA and SERCA rates is much smaller in the Wang

et al. model than in our model, while it is the contrary for the ratio between the IPR and SERCA rates. The equilibrium Ca^{2+} concentrations of the two models are also compared in Table S1.

quantity	expression	this work	Wang *et al.* [4]	ratio
J_{SOCC}/J_{PMCA}	$\frac{V_s}{K_s}\frac{K_p}{V_p}$	0.00628	0	0
J_{leakin}/J_{PMCA}	$\frac{\alpha_0}{V_p}$	0	0.0111	/
J_{ROCC}/J_{PMCA}	$\alpha_1\frac{K_p}{V_p}$	0.000209	0.0889	424
J_{VOCC}/J_{PMCA}	$\frac{V_s}{K_s}\frac{K_p}{V_p}$	0	0.2	/
J_{PMCA}/J_{SERCA}	$\frac{V_p}{K_p}\frac{K_e}{V_e}$	0.1	0.0125	0.125
J_{IPR}/J_{SERCA}	$k_{IPR}\frac{K_e}{V_e}$	0.0133	0.123	9.25
J_{RyR}/J_{SERCA}	$k_{RyR}\frac{K_e}{V_e}$	0	0.0371	/
J_{leakSR}/J_{SERCA}	$J_{SR}\frac{K_e}{V_e}$	0.0002	0.00222	11.1
J_{leakSR}/J_{IPR}	$\frac{J_{SR}}{k_{IPR}}$	0.015	0.018	1.2
resting $[Ca^{2+}]_i (\mu M)$	c^*	0.0681	0.161	N.A.
resting $[Ca^{2+}]_{SR} (\mu M)$	c_s^*	158	27.4	N.A.

Table S1. Relative magnitudes of the different Ca^{2+} fluxes in our model and in the Wang *et al.* model [4]. The last column gives the ratio of the previous two. The equilibrium Ca^{2+} concentrations are also given.

References

1. Demaurex N, Frieden M (2003) Measurements of the free luminal ER Ca2+ concentration with targeted cameleon fluorescent proteins. Cell Calcium 34: 109–119.

2. Tang Y, Stephenson JL, Othmer HG (1996) Simplification and analysis of models of calcium dynamics based on IP3-sensitive calcium channel kinetics. Biophys J 70: 246–63.

3. Sneyd J, Tsaneva-Atanasova K, Yule DI, Thompson JL, Shuttleworth TJ (2004) Control of calcium oscillations by membrane fluxes. PNAS 101: 1392–6.

4. Wang IY, Bai Y, Sanderson MJ, Sneyd J (2010) A mathematical analysis of agonist- and KCl-induced Ca2+ oscillations in mouse airway smooth muscle cells. Biophys J 98: 1170–81.
